# FACTORS ASSOCIATED WITH MEALTIME LANGUAGE CHARACTERISTICS IN NURSING HOME STAFF AND RESIDENTS WITH DEMENTIA

**DOI:** 10.1093/geroni/igac059.1077

**Published:** 2022-12-20

**Authors:** Wen Liu, Ying-Ling Jao, Si On Yoon

**Affiliations:** University of Iowa, Iowa City, Iowa, United States; Pennsylvania State University, University Park, Pennsylvania, United States; The University of Iowa, Iowa City, Iowa, United States

## Abstract

Interactive staff-resident communication is crucial for nursing home residents with dementia requiring mealtime assistance. Effective communication may facilitate food intake and promote function and nutrition. However, understanding of staff and resident language characteristics in mealtime interactions is limited. This study examined language characteristics and associated factors in mealtime interactions. This was a secondary analysis using data from videotaped mealtime observations (N=160) involving 36 staff and 27 residents with moderate-to-severe dementia (53 staff-resident dyads) in 9 nursing homes. The dependent measures were 1) the number of words produced in each utterance (expression length), and 2) whether staff named the resident in each utterance (naming the resident). Mixed-effects models examined the effect of utterance quality (positive vs. negative utterances), intervention (pre- vs. post-communication training), and subject speaking (staff vs. resident), adjusting resident comorbidities and dementia stage. Staff (mean=4.30, SD=2.98) produced significantly longer utterances than residents (mean=2.64, SD=2.27). Expression length was modulated by utterance quality and intervention. Staff’s negative utterances were shorter than their positive utterances, while residents’ negative utterances were longer than their positive utterances. Staff’s negative utterances became longer after the intervention while the length of positive utterances remained similar pre- and post-intervention. Staff named the resident in 16.72% of their utterances and was more likely to name residents with severe dementia. Findings emphasize the potential benefit of communication training on mealtime interactions. Findings also highlight the need to further examine the impact of language characteristics on food intake, which may guide intervention development to promote nutrition for residents with dementia.

